# Notes of Cases

**Published:** 1863-06

**Authors:** 


### Editorial.

---

### NOTES OF CASES

I. Mrs. I., aged 30, had a number of teeth affected with chronic *periostitis* and *exostosis*. She had sufferred considerably for years, with (so-called) neuralgia. Occasionally there was a purulent discharge from one of her ears; and her hearing was so defective that it was quite difficult to converse with her. About the first of May, I removed nine diseased teeth from the upper jaw, free hemorrhage taking place. The next day but one, she heard the striking of the family clock, a sound not heard for years before. A month after the operation, she returned, able to hear ordinary conversation across a room. To all appearance, the sense of hearing is perfectly restored. With the facts connected with the early history of her loss of hearing, I am not familiar.

II. Miss C., aged 18, of sanguino-lymphatic temperament, called to have fourteen upper teeth extracted. From her appearance, I was led to suspect that her menstrual period was nearly approaching, but inquiring of her, she stated that nearly two weeks would elapse befor her regular time. The next morning was set for the operation, and she was instructed not to eat any breakfast. The appointment was kept, and she was brought thoroughly under the influence of chloroform,—no ether combined with it. Complete anasthesia was produced with difficulty; but nothing occurred during the operation to cause any uneasiness. The teeth and alveolar processes were excessively developed. The operation would have been very severe without an anasthetic. About the usual amount of hemorrhage occurred An hour after the operation, the patient walked, with a friend, the distance of two squares, to her residence. The fourth day after the operation, the gums began to bleed. The hemorrhage was controlled with difficulty. The persulphate of iron and pressure were mainly relied on for the time. A distant relative of the patient had died from hemorrhage of the gums, consequently both patient and friends were greatly frightened. I now learned tha the patient was subject to vicarious menstruation, hemorrhage from the nose frequently taking the place of the natural flow, and that, notwithstanding the patient's statement before the operation, this was the proper time for the menstrual paroxysm. I prescribed one grain of opium with four of acetate of lead, to be taken every three hours, until the anodyne effects of the opium were observable. In this way the hemorrhage was promptly controled. The family physician was called to see the case, and cordially approved the treatment. The recovery was satisfactory.

III. Mrs. B., aged 32, of nervous temperament, and delicate health, had nine teeth to extract. A number of them had been dressed off for the purpose of fitting a plate over them. The plate, not proving satisfactory, was soon abandoned, and the remaining teeth gradually approximated each other, till the roots referred to were firmly held between them. I administered chloroform, in the presence of the family physician, producing full anasthesia. When the second tooth was removed, the breathing suddenly ceased. Artificial respiration was resorted to for a few seconds, when normal breathing was re-established, and the operation was rapidly completed. The recovery was natural and normal; and the general health of the patient is much improved.

IV. Mrs. ———, the wife of a physician, had nine teeth to remove. She was familiar with the action of chloroform, and was not afraid of it. In six minutes from the time she took a seat in the operating chair, she was lying on a sofa, in the office parlor, conversing naturally with her sister, the nine teeth having been removed while she was totally unconscious.

V. Master John H., aged 8, a pale delicate boy, had an abcess formed at the root of a lower first permanent molar. The constitution was greatly disturbed, and the tooth much decayed. I determined to extract it. The operation was borne heroically. The operation was performed at four in the afternoon. The hemorrhage soon abated. In the morning his pillow was saturated with blood, and I was sent for. The bleeding was promptly arrested by the persulphate of iron and a compress; but the application had to be continued some days.

Remarks.—The first case noted above is *practically* instructive. In treating diseases of the ear, the condition of the teeth should always be noticed. In this case, an intellectual, sprightly woman was almost lost to society for years; and this view of the case is serious enough, without taking into the account her sufferings and impaired health.

The second case shows the importance of knowing the exact condition of the patient before performing a severe operation. Had I known that her menstrual period was but four days hence, I would have postponed the operation, and, especially, if I had known that she was subject to vicarious menstruation. But she wanted new teeth at a given time, and hence her deception.

The fourth case demonstrates the necessity of presence of mind. Without artificial respiration, I have but little doubt that the case would have proved fatal. Had I followed my own judgment fully in this case, I would have brought her more rapidly under the influence of the chloroform than was done, to avoid the inhalation of so large a quantity; but I yielded considerably to the judgment of the *family* physician who was of course better acquainted with her constitution. When the family physician is a gentleman and a *physician*, it is usually dangerous to differ with him.

The fourth case only makes us wish that anasthetics would always act that way.

The fifth case suggests nothing, unless it be the use of the persulphate as a styptic. The perchloride of iron, however, I regard as preferable. Some day I will suggest a simple process for making it. W.

